# Multi-Omics Profiling Reveals Phenotypic and Functional Heterogeneity of Neutrophils in COVID-19

**DOI:** 10.3390/ijms25073841

**Published:** 2024-03-29

**Authors:** Lin Zhang, Hafumi Nishi, Kengo Kinoshita

**Affiliations:** 1 Tohoku Medical Megabank Organization, Tohoku University, Sendai 980-8573, Miyagi, Japan; 2 Department of Applied Information Sciences, Graduate School of Information Sciences, Tohoku University, Sendai 980-8579, Miyagi, Japan; 3 Faculty of Core Research, Ochanomizu University, Tokyo 112-8610, Japan; 4 Advanced Research Center for Innovations in Next-Generation Medicine, Tohoku University, Sendai 980-8573, Miyagi, Japan; 5 Department of In Silico Analyses, Institute of Development, Aging and Cancer (IDAC), Tohoku University, Sendai 980-8575, Miyagi, Japan

**Keywords:** neutrophil phenotypes and function, cellular deconvolution, heterogeneity, multi-omics, COVID-19

## Abstract

Accumulating evidence has revealed unexpected phenotypic heterogeneity and diverse functions of neutrophils in several diseases. Coronavirus disease (COVID-19) can alter the leukocyte phenotype based on disease severity, including neutrophil activation in severe cases. However, the plasticity of neutrophil phenotypes and their relative impact on COVID-19 pathogenesis has not been well addressed. This study aimed to identify and validate the heterogeneity of neutrophils in COVID-19 and evaluate the functions of each subpopulation. We analyzed public single-cell RNA-seq, bulk RNA-seq, and proteome data from healthy donors and patients with COVID-19 to investigate neutrophil subpopulations and their response to disease pathogenesis. We identified eight neutrophil subtypes: pro-neutrophil, pre-neutrophil, immature neutrophil, and five mature neutrophil subpopulations. The subtypes exhibited distinct features, including diverse activation signatures and multiple enriched pathways. The pro-neutrophil subtype was associated with severe and fatal disease, while the pre-neutrophil subtype was particularly abundant in mild/moderate disease. One of the mature neutrophil subtypes showed consistently large fractions in patients with different disease severity. Bulk RNA-seq dataset analyses using a cellular deconvolution approach validated the relative abundances of neutrophil subtypes and the expansion of pro-neutrophils in severe COVID-19 patients. Cell–cell communication analysis revealed representative ligand–receptor interactions among the identified neutrophil subtypes. Further investigation into transcription factors and differential protein abundance revealed the regulatory network differences between healthy donors and patients with severe COVID-19. Overall, we demonstrated the complex interactions among heterogeneous neutrophil subtypes and other blood cell types during COVID-19 disease. Our work has great value in terms of both clinical and public health as it furthers our understanding of the phenotypic and functional heterogeneity of neutrophils and other cell populations in multiple diseases.

## 1. Introduction

Coronavirus disease (COVID-19), caused by severe acute respiratory syndrome coronavirus 2 (SARS-CoV-2), can alter the leukocyte phenotype in severe cases, including alterations in the immune system, exhaustion of lymphocytes, and changes in hematopoiesis [1,2,3,4,5,6]. In addition to macrophage, B-cell, and T-cell activation, numerous studies have shown neutrophil activation by virus infections [2,7,8,9,10,11]. A recent study identified alterations in neutrophil phenotypes that may be associated with the pathogenesis of several diseases [12]. However, the plasticity of neutrophil phenotypes and their relative impact on COVID-19 pathogenesis have not been well described.

Neutrophil populations in health are characterized by the variability in their phenotypes and functions when mobilized into different sites. Specifically, bone marrow neutrophils, originating from granulocyte–monocyte progenitors, differentiate into several major types: pro-neutrophils, pre-neutrophils, immature neutrophils, and mature neutrophils, while blood neutrophils predominately consist of mature neutrophils undergoing circadian aging [13]. In contrast to mature neutrophils, immature neutrophils are generally programmed to stay in bone marrow rather than entering circulation [13,14,15]. However, under stress conditions such as inflammatory stimuli, neutrophil phenotypes and functions of neutrophils can be rapidly altered. Immature neutrophils, similar to mature neutrophil compartments, can also be mobilized into the blood and recruited to inflammation sites [13]. Over the past five years, accumulating evidence has revealed unexpected phenotypic and functional heterogeneity of neutrophils in multiple diseases, including infections, autoimmunity, sterile injury, cancers, atherosclerosis, and type-2 diabetes [12]. For example, three subsets of neutrophils, namely mature high-density neutrophils, mature low-density neutrophils (LDNs), and immature LDNs, have been observed in the circulation of both patients with cancer and tumor-bearing mouse models [16,17,18,19]. A study of 124 patients and eight mice revealed the heterogeneity of neutrophils in liver cancer, and some of the subpopulations were found to be associated with an unfavorable prognosis, where the CCL4+ neutrophil population can recruit macrophages and the PD-L1+ population displays suppression of T-cell cytotoxicity [20]. Furthermore, neutrophil subpopulations have been identified to have opposing immune functions in some contexts, such as collateral damage during post-injury inflammation, compared with the regeneration of post-injury tissue [21,22,23,24]. Several other anti-inflammatory functions, including the suppression of T-cell function and the release of α-defensins to inhibit macrophage-driven inflammation, have also been described [12]. Although the role of neutrophils in viral infections remains unclear, increasing evidence has demonstrated that neutrophils contribute to resolving viral infection [25]. In human circulating neutrophils, genes related to antiviral defense are specifically expressed [26,27]. Further research has revealed that gene expression profiles in basal neutrophils vary significantly among human donors compared to monocytes or lymphocytes, and genes with high variability were predominantly associated with immune functions such as antiviral responses [13,28,29]. In a mouse model, depletion of neutrophils with an anti-Gr-1+ antibody caused severe diseases, such as respiratory dysfunction and dysregulated immunity during influenza A virus (IAV) infection [30,31]. Literature on viral respiratory diseases (VRDs) provides an understanding of neutrophil heterogeneity and functions in several stages, such as heterogeneous neutrophil infection, development, and activation [25]. In the infection stage, IAV-infected neutrophils produce less human cathelicidin LL-37 and can act as antigen-presenting cells for antiviral CD8+ T cells [32,33]. In the development stage, high fractions of immature neutrophils, which were not affected by bacterial coinfection, were observed in infants with various VRDs [34]. In the activation stage, a degranulated form of neutrophils (CD16Int low-density neutrophil population) contributes to immunosuppression in VRDs and acute respiratory distress syndrome [35].

**Figure 1:**
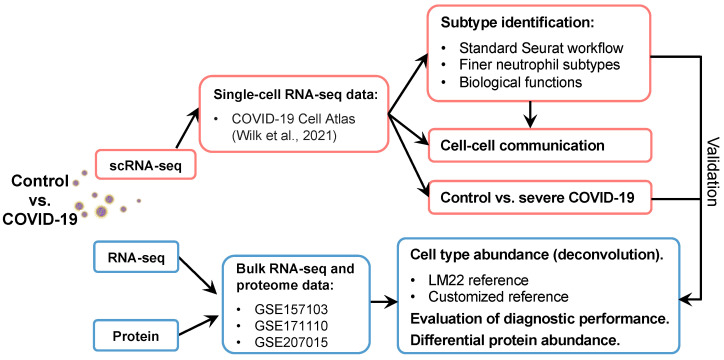
Workflow of this study. The scRNA-seq dataset was downloaded from the COVID-19 Cell Atlas [11]. Neutrophils were used for further analyses, including finer subtype identification and cell–cell interactions. The disease and control groups were compared to investigate the mechanisms underlying disease pathogenesis. Finally, the results obtained from scRNA-seq data were validated using public bulk RNA-seq and proteome data.

Discoveries of the versatile phenotypes and functions of neutrophils have opened new doors to understand their contributions to antimicrobial functions as well as homeostatic and pathogenic immune processes [36]. The present study aimed to analyze scRNA-seq, bulk RNA-seq, and plasma proteome data of healthy individuals and patients with COVID-19 to identify and validate the heterogeneity of neutrophils. This study further investigated the alterations in phenotypes and contributions in response to disease severity for each neutrophil subpopulation. The workflow used for the neutrophil subpopulation investigation and validation is shown in Figure 1. We observed that neutrophils were activated after SARS-CoV-2 infection in the scRNA-seq and bulk RNA-seq datasets. First, the standard Seurat workflow was used to identify finer subtypes of neutrophils and detect the activation signatures of each subtype [37]. Biological functions and cell–cell communication analyses were subsequently used to describe the phenotypic and functional heterogeneity of each subtype. Comparisons between severe COVID-19 and healthy cells were performed to reveal the mechanisms underlying COVID-19 progression, including differentially expressed gene (DEGs) identification, gene regulatory network, pathway enrichment, and differential protein abundance analyses. Next, the subtypes identified using scRNA-seq data were passed on to public bulk RNA-seq datasets to investigate cell type abundance using the cellular deconvolution approach in terms of COVID-19. Of note, two references, that are modified LM22 and customized references, were used for cell type abundance analysis. Several predictors have been evaluated for their diagnostic performance in predicting severe disease. Our work holds great value for both clinical and public health for understanding the heterogeneity of neutrophils and other cell populations in multiple diseases.

## 2. Results

### 2.1. Characterization of the scRNA-seq Data

Neutrophil scRNA-seq data were obtained from 40 blood samples of healthy individuals and patients with COVID-19 [11], including 23 males and 17 females, and aged 18 to 80+ years (Table S1). This dataset was a neutrophil subset of the scRNA-seq dataset in the original study, which revealed a total of 174,753 cells belonging to 15 cell types (Figure S1). The extracted neutrophil dataset, including 39,845 cells, was used to study their phenotypic and functional heterogeneity. Based on the embedding dimension reduction of the source data, we visualized the obtained neutrophils on a UMAP plot in terms of disease severity and observed that some neutrophil subpopulations showed different abundances in terms of COVID-19 severity, suggesting that neutrophils are heterogeneous and that phenotypes with potentially different functions under physiological and pathological conditions need further investigation (Figure S1).

### 2.2. Identification and Description of Neutrophil Subpopulations

**Figure 2:**
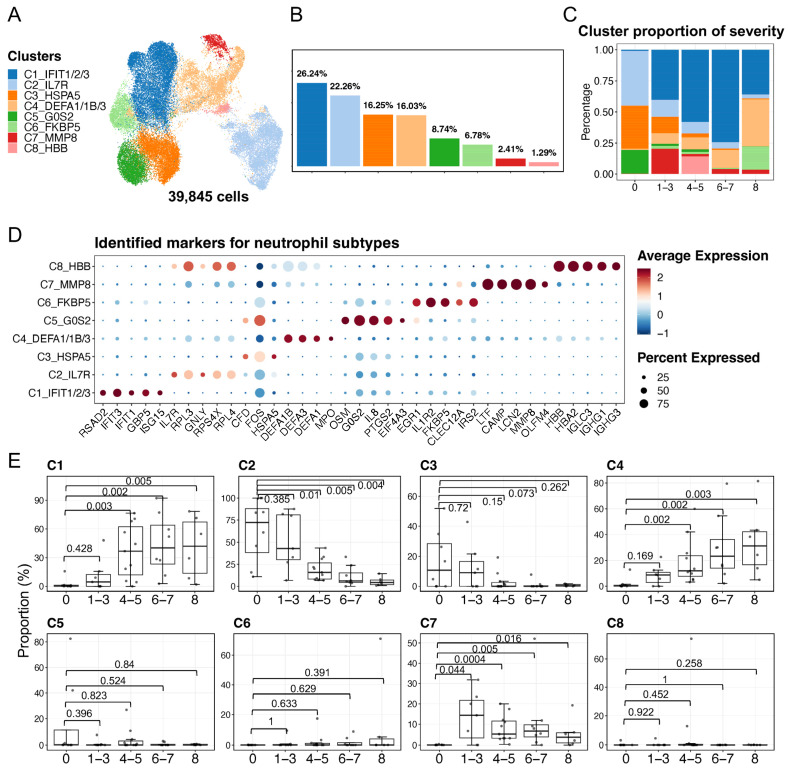
Characterization of neutrophil heterogeneity. (**A**) The UMAP plot exhibits the eight identified neutrophil subtypes. (**B**) The bar plot indicates the cell proportions in each subtype. (**C**) The bar plot shows subtype compositions across the severity of COVID-19. The color scheme was shared among (**A**–**C**). (**D**) Dot plots represent the identified top five markers for each subtype that were identified by using the FindAllMarkers in Seurat v4. (**E**) Proportions of each neutrophil subtype in each donor. The x axes correspond to the COVID-19 severity of each donor. Shown are exact *p* values that were calculated by a two-sided Wilcoxon rank-sum test. The WHO scores were used to classify healthy (WHO score 0), mild (WHO score 1–3), moderate (WHO score 4–5), severe (WHO score 6–7), and fatal (WHO score 8) donors.

Using the scRNA-seq data of neutrophils, we identified finer subtypes and described their heterogeneous functions. First, the standard Seurat workflow for scRNA-seq data processing was used to identify finer neutrophil subtypes. Together with the visualization of cell movements at multiple resolutions of clustering using Clustree (Figure S2A) [38], eight neutrophil subtypes, namely C1–C8, were determined based on the selected resolution (Figure 2A,B). In addition, we utilized ROGUE, an R package (version 4.1.1), for assessing the quality of the eight identified neutrophil subtypes [39]. As a result, the low ROGUE value (approximately 0.8) for the overall neutrophils (consisting of 39,845 cells) demonstrated their heterogeneity prior to the finer clustering, whereas the relatively high ROGUE values of each subtype post-clustering (the majority of median values exceeded 0.9) suggested the high purity of the identified neutrophil subtypes (Figure S2B). We next calculated the compositions of the eight identified subtypes in terms of COVID-19 severity, suggesting that subtype compositions varied according to the disease severity (Figure 2C). Specifically, the C1 subtype exhibited the highest fraction in patients with mild, moderate, and severe disease, whereas the C2, C3, and C5 subtypes were abundant in healthy individuals. The C4 subtype was particularly prevalent among severely ill and fatal donors. The C6, C7, and C8 subtypes also exhibited some degree of extensions in patients with COVID-19. The activation signatures of the eight neutrophil subtypes were detected, and then some of the subtype-specific signatures were considered as markers for the subtypes (Table S2, Figure S3A). The neutrophil subtypes were renamed by adding the subtype-specific gene symbols *IFIT1/2/3* (C1), *IL7R* (C2), *HSPA5* (C3), *DEFA1/1B/3* (C4), *G0S2* (C5), *FKBP5* (C6), *MMP8* (C7), and *HBB* (C8) (Figure 2A). Furthermore, we identified that these subtypes represent neutrophils at various developmental stages. The expressions of the top five identified markers (Figure 2D) and the markers from the literature (Figure S3B) suggested that three of the identified neutrophil subtypes in the present study were immature neutrophils, while the other five subtypes were mature neutrophils. Differential expressions of *CXCR2*, *MME*, and *CD16* enable discrimination of immature neutrophils (CXCR2–MME–CD16mid; which are the C4, C7, and C8 subtypes) and mature neutrophils (CXCR2+MME+CD16hi; which are the C1–C3, C5, and C6 subtypes). Among the above markers, defensin genes (*DEFA1/1B/3*) and myeloperoxidase (*MPO*) were detected in the C4 subtype, whereas lactoferrin (*LTF*), gelatinase (*MMP8*), cathelicidin (*CAMP*), and integrin (*ITGAM*) were observed in the C7 subtype, suggesting that the C4 was associated with azurophilic/primary granules (that is, pro-neutrophils) and C7 with specific/secondary and gelatinase/tertiary granules (that is, pre-neutrophils) [40,41,42]. In addition, the transcriptome profiling in neutrophils among human donors shows vast differences [28], leading us to quantify the proportions of the identified neutrophil subtypes for each donor in relation to COVID-19 severity (Figure 2E). We observed significant increases in the C1, C4, and C7 subtypes in COVID-19 patients, while C2 was significantly depleted in varying severities of the disease, except in mild cases.

**Figure 3:**
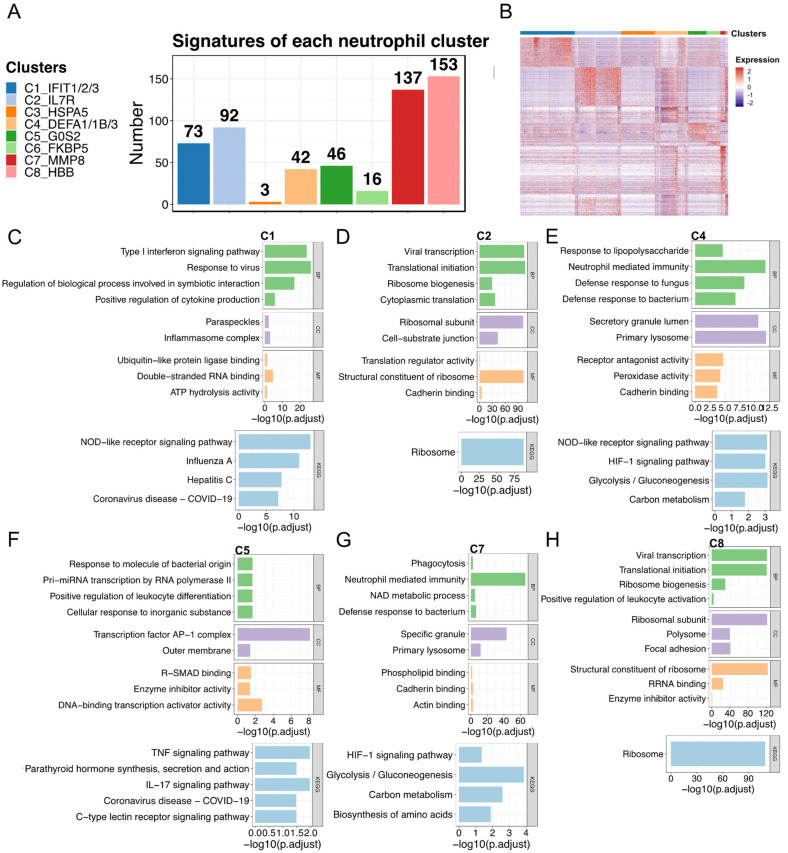
Biological functions of each neutrophil subtype. (**A**) Gene signatures of the eight subtypes. (**B**) The heatmap represents the signature expression in (**A**). The identified signatures in (**A**) were used for enrichment analysis. (**C**–**H**) The representative enriched GO and KEGG pathways of each subtype are presented: (**C**) C1, (**D**) C2, (**E**) C4, (**F**) C5, (**G**) C7, and (**H**) C8. The representative pathways of C3 and C6 are not provided owing to the small size of identified signatures. GO enrichment analysis was performed based on BP, CC, and MF. GO, Gene Ontology; KEGG, Kyoto Encyclopedia of Genes and Genomes; BP, biological process; CC, cellular component; MF, molecular function. Collectively, the present study illustrated neutrophil plasticity in phenotypes and functions under physiological and pathological conditions. Some immature neutrophil subtypes (e.g., C4 and C7), normally mobilized into bone marrow under steady-state conditions, were released into the circulation after SARS-CoV-2 infection. Together with specific mature neutrophil subtypes (e.g., C1), these characterized immature and mature neutrophil subtypes may jointly contribute to the immune response against viral infection. Oppositely, some mature neutrophil subtypes (e.g., C2) were drained during COVID-19 progression.

We next analyzed the functions of the signatures of each subtype. The number of signatures varied considerably among subtypes (Figure 3A). Compared with most of the C7 (*n* = 137) and C8 (*n* = 153) genes, only a few signatures were detected in the C3 (*n* = 3) and C6 (*n* = 16) subtypes. Next, we visualized the expression of the signatures and performed GO and KEGG pathway enrichment analyses to characterize the functions of each subtype (Figure 3B–H). The enriched pathways of the C3 and C6 subtypes were not detected because of the small number of signatures; thus, their related functions could not be described. We found that the subtypes displayed unique or commonly enriched pathways, which may lead to different phenotypes or contribute to different conditions (i.e., healthy and COVID-19). In detail, C2, C5, and C8 were associated with the ribosome pathway and transcription activator activity, while C1, C4, and C7 were associated with neutrophil-mediated immunity, glycolysis/gluconeogenesis, the NOD-like receptor signaling pathway, and the HIF-1 signaling pathway. Notably, the type I interferon signaling pathway was significantly enriched in C1. Previous studies on systemic lupus erythematosus (SLE) showed that neutrophils in patients induced type I interferon production in plasmacytoid dendritic cells, and one possibility based on this is that the presence of interferons in patients with SLE may release immature neutrophils [13,43,44]. Combining our findings with consistently large fractions of C1 (mature neutrophils) in patients under diverse severity of COVID-19 and existing immature neutrophils during disease (Figure 2 and Figure S3), we here speculated that the C1 that associated with the type I interferon signaling pathway may also potentially contribute to the release of immature neutrophils.

### 2.3. Diverse Cell–Cell Communications among Neutrophil Subtypes and Other Blood Cell Types

**Figure 4:**
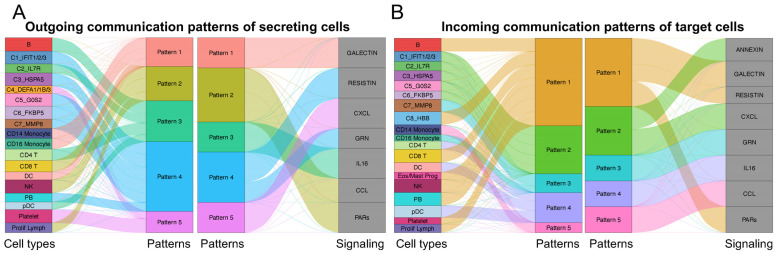

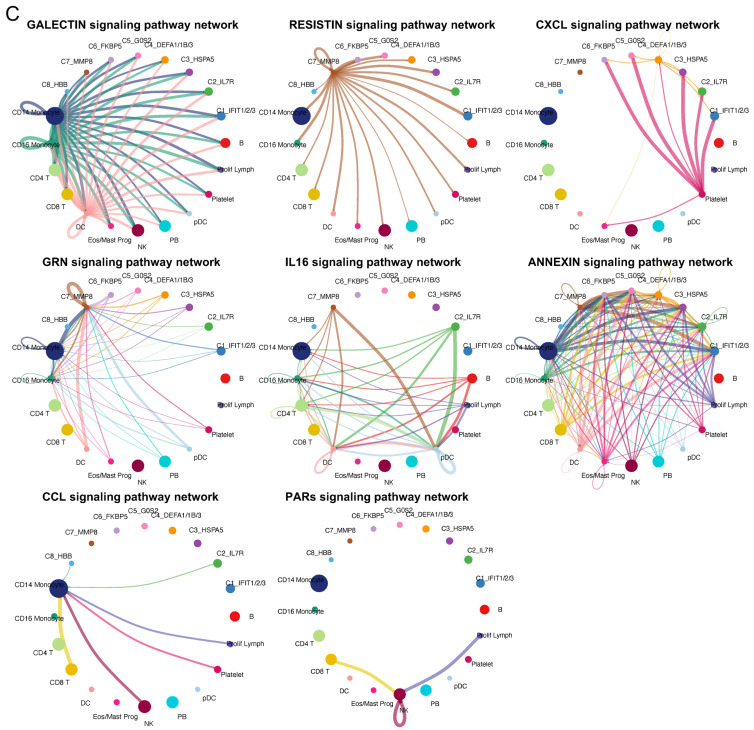
Cell–cell communications among the eight identified neutrophil subtypes and other cell types. Cell–cell communications (CCCs) among neutrophil subtypes and other cell types were performed by focusing on ligand–receptor (L–R) pairs. The (**A**) outgoing and (**B**) incoming communication patterns are represented for each cell type. Alluvial plots were used to visualize the cell types, inferred latent patterns, and associated signaling pathways. Flow thickness refers to the degree of contribution of cell types or signaling pathways to the latent patterns. (**C**) Networks indicate the CCCs of representative signaling pathways between neutrophil subtypes and others. Cell types are represented by different colors. The size of the circle is proportional to the cell number. The edge width represents the number of L–R pairs. Arrows represent the direction of interactions from one cell (sender) to another (receiver).

Next, we described the phenotypes and functions of the eight identified neutrophil subtypes by cell interaction analysis to understand how neutrophil subtypes interact with other cell types, such as monocytes, B-cells, and T-cells. We analyzed cell–cell communications (CCC) using the CellChat (version 1.6.1) tool to identify representative ligand–receptor (L–R) pairs. The eight neutrophil subtype labels (C1–C8) were transferred to the original scRNA-seq data, which included 15 cell types. We inferred the signaling pathways and communication patterns and then visualized the representative signaling pathways in the networks (Figure 4). Five outgoing and incoming communication patterns were identified between cell types and their associated signaling pathways. Outgoing communication patterns indicate how secreting cells (acting as senders) drive interactions via specific signaling pathways by secreting ligands. In contrast, incoming communication patterns demonstrate how target cells (acting as receivers) contribute to interactions by expressing their receptors. Regarding the results of both the outcoming and incoming patterns in terms of cell types, we found that neutrophil subtypes could be classified into various patterns connected to a total of eight representative signaling pathways, suggesting that the identified pathways can have different roles as senders or receivers depending on the subtypes to which they pertain. We next visualized six of the identified signaling pathways in the networks (Figure 4C). We found that the roles (i.e., senders and receivers) of the neutrophil subtypes may vary for each signaling pathway. For instance, all eight subtypes received signals from monocytes or dendritic cells in the GALECTIN signaling pathway, whereas only the C7 subtype sent signals to all other cell types in the RESISTIN signaling pathway.

**Figure 5:**
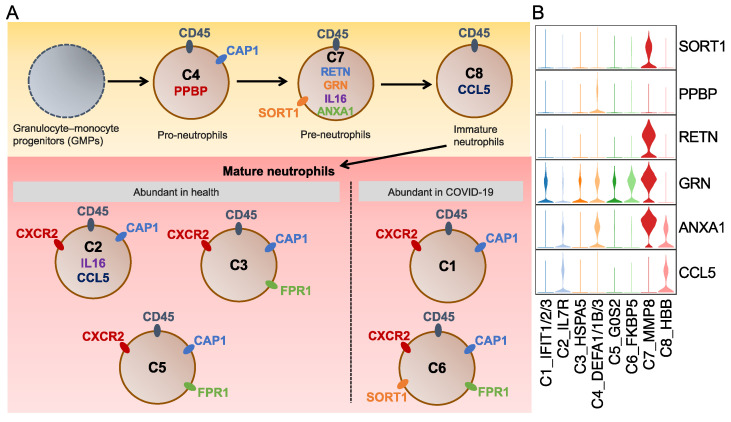
Schematic diagram for the neutrophil phenotypes. (**A**) Schematic diagram for the eight identified neutrophil subtypes. Ligands and receptors for each subtype are shown inside and outside the cell, respectively. The same color marks the shared ligands and receptors among subtypes. (**B**) Violin plots represent the expression of selected ligands and receptors. The selected genes were identified to be upregulated in the relative neutrophil subtypes by using the FindAllMarkers in Seurat.

To sum up, we created a diagram showing L–R pairs in these signaling pathways and visualized their expression among the neutrophil subtypes (Figure 5 and Figure S4). The diagram reveals six ligands (*RETN*, *GRN*, *IL16*, *ANXA1*, *CCL5*, and *PPBP*) and five receptors (*SORT1*, *CXCR2*, *CD45*, *CAP1*, and *FPR1*). Some of them were further identified differential upregulation in the specific neutrophil subtypes via FindAllMarks in Seurat, but the others showed no such different regulation. Among the differentially expressed ligands and receptors, *SORT1*, *RETN*, and *GRN* were specific to the C7 subtype, and the PPBP ligand (pro-platelet basic protein) of the CXC chemokine family was unique to the C4 subtype (Figure 5B and Table S2). A previous study on PPBP reported that a subpopulation of neutrophils with a high *PPBP* level may correspond to neutrophil–platelet aggregates and, therefore, could serve as a promising blood-based prognostic biomarker for solid tumors, offering clinical value in their assessment [45]. Unlike subtype-specific genes, *CCL5* was common in the C2 and C8 subtypes, and Annexin A1 (*ANXA1*) was observed in the C4 and C7 subtypes. A previous study reported that the *ANXA1* expression in neutrophils can recruit monocytes, leading to the clearance of remaining neutrophils in the resolution of inflammation [46].

### 2.4. Neutrophil Profiling Was Altered under Physiological and Pathological Conditions

**Figure 6:**
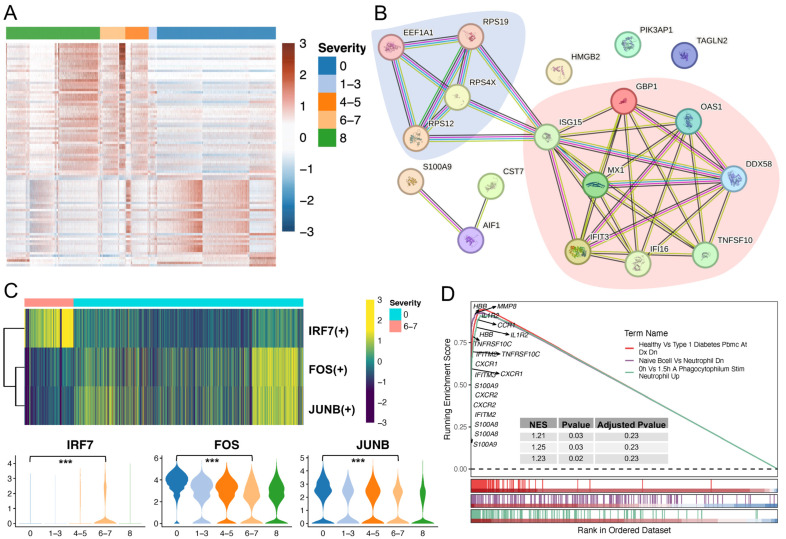
Comparisons of healthy individuals and patients with severe COVID-19. (**A**) The heatmap shows the DEGs in severe COVID-19 when comparing to healthy individuals. Five WHO severity score groups: healthy (0), mild (1–3), moderate (4–5), severe (6–7), and fatal (8). (**B**) Functional protein association network analysis using the STRING database. Overlaps of DEGs from scRNA-seq and plasma proteome datasets were used. Blue and red shades represent the two main networks: SARS-CoV-1/2 modulates host translation machinery and immune system, respectively. (**C**) Selected differentially expressed TFs. Selected key TF modules based on the pySCENIC (upper) and FindMarkers in Seurat analyses (bottom) by comparing severe to healthy cells. Significant gene expression was determined by comparing the severe (6–7) and healthy (0) groups. *** *p* value < 0.001. (**D**) GSEA analysis was performed based on the C7_MMP8 immunologic signature gene sets from MSigDB. GSEA: gene set enrichment analysis.

To explore how neutrophils drive inflammation during COVID-19 progression, we compared severe COVID-19 to healthy neutrophils and identified 55 upregulated and 38 downregulated genes (Table S3, Figure 6A and Figure S5A). In contrast to the enriched ribosome pathway using downregulated genes, upregulated genes were associated with influenza response and certain signaling pathways, such as the NOD-like receptor, RIG-I-like receptor, *IL-17* receptor, and Toll-like receptor signaling pathways (Figure S5B). Several studies have demonstrated the important roles of these signaling pathways in either innate immunity or COVID-19 progression, including their utility as diagnostic biomarkers and potential treatment strategies [47,48,49,50,51,52,53]. We next performed significantly differential protein abundance analysis by comparing healthy and severe/critical samples from the plasma proteome dataset, finding an overlap of 18 genes consistently either upregulated (12 genes) or downregulated (six genes) compared to the scRNA-seq data (Figure S6). These genes were used to examine functional protein association networks via the STRING database [54]. Two main networks were revealed: SARS-CoV-1/2 modulates host translation machinery (downregulated genes) and immune system (upregulated genes) (Figure 6B). In addition, we found that some TFs were differentially expressed in severe COVID-19, including TF upregulations of *IRF7*, *SP100*, *HMGB2*, and *BCL6*, and downregulations of *FOS* and *JUNB* (Table S3). We also investigated TF regulon changes and evaluated their activities via pySCENIC and found that some of the inferred TFs displayed an overlap with the DEGs shown in Table S3. Specifically, the IRF7 (+) regulon was activated in severe COVID-19, whereas FOS (+) and JUNB (+) regulons were activated in healthy neutrophils (Figure 6C). We also performed GSEA based on the transcriptome of patients with severe disease and found that 971 gene sets were enriched with a *p*-value cutoff of 0.05 (Table S4; Benjamini–Hochberg method was used to adjust the *p*-value). We visualized three representative enriched gene sets and marked multiple S100 family members (e.g., *S100A8/A9*), interferons (e.g., *IFITM2*), and *MMP* family members (e.g., MMP8), as well as cytokines, chemokines, and their receptors (e.g., *IL1R2* and *CXCR1*) (Figure 6D). The selected genes showed high expression levels in the severe COVID-19 transcriptome.

Recent studies have identified virus-induced senescence as the underlying cause of cytokine release and inflammation in patients with COVID-19, and a robust upregulation of the senescence phenotype in the majority of COVID-19 patients was particularly observed in lung epithelial cells after viral infection [55,56,57]. Considering these studies, we investigated cell senescence status across the eight neutrophil subtypes using three senescence-associated gene sets (Figure S7). Some of the neutrophil subtypes abundant in COVID-19 (e.g., C2, C6, and C7) showed high levels of senescent cells, whereas others (C4 and C8) exhibited low levels. Our findings indicate that cellular senescence may not be commonly triggered in SARS-CoV-2-infected neutrophils compared to that in infected lung epithelial cells, and that senescence induction is dependent on the subpopulations of neutrophils.

### 2.5. Validation of C4 Neutrophil Subtype Abundance and Evaluation of Predictors towards Severe COVID-19

**Figure 7:**
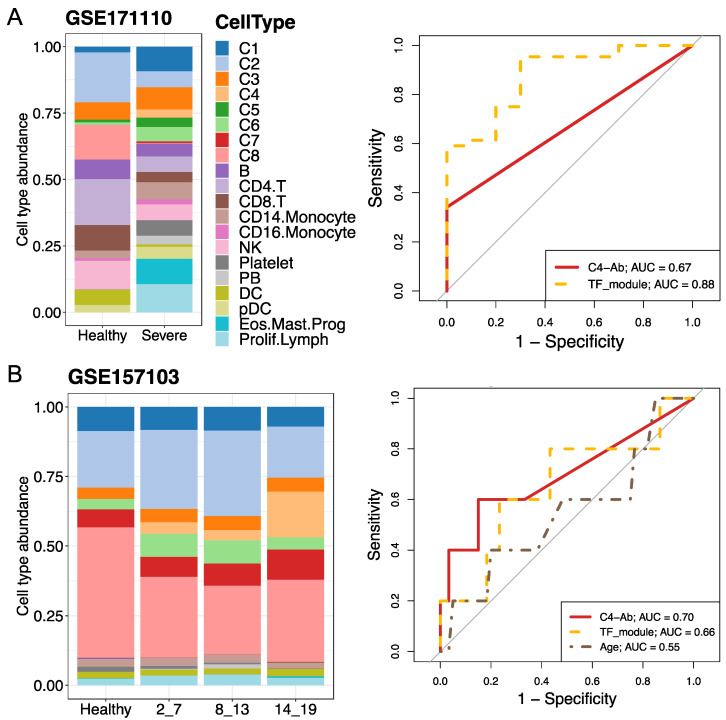
Cell type abundance estimation and severe predictions using bulk RNA-seq and human plasma proteome datasets. (**A**,**B**) Analyses were performed using GSE171110 and GSE157103, respectively. Cellular deconvolution analysis was performed using the customized reference based on the scRNA-seq dataset. The bar plots indicate the eight subtype proportions between healthy individuals and patients with COVID-19. The average subtype fractions for control–case groups were used. In the case of GSE157103, SOFA scores varying from 2 to 19 were used to evaluate disease severity. For GSE171110, healthy and severely ill patients were analyzed. The right panels represent disease predictions for multiple predictors using the area under the receiver operating characteristic (ROC) curve (AUC). Predictors included C4-Ab (abundance of neutrophil subtype C4), specific differentially expressed gene sets (namely the TF module, including IRF7, FOS, and JUNB.) in severe cases, and/or age. The relative expression levels of these genes in the scRNA-seq dataset are shown in Figure 6C. SOFA: sequential organ failure assessment.

After investigating the phenotypes and functions of the identified neutrophil subtypes and the mechanisms of COVID-19, we then used two bulk RNA-seq datasets (accession numbers GSE171110 and GSE157103) to validate our findings from the scRNA-seq data and evaluate the performance of severe COVID-19 prediction models. On the one hand, cellular deconvolution analysis using a modified LM22 expression signature reference indicated that the relative abundances of neutrophil subpopulations were increased in severe COVID-19 patients for the two bulk RNA-seq datasets (Figure S8A). On the other hand, the signature matrix of the identified eight neutrophil subtypes (including immature and mature neutrophils) and other main blood cell types was used as a customized reference to investigate their relative abundances. We observed that the C4 fraction (pre-neutrophils) increased along with the SOFA score or severe disease that was similar to an expansion in severe and fatal cases from the scRNA-seq data (Figure 7). In addition, we calculated the Pearson correlation between the fraction of each neutrophil subtype and the SOFA score using the GSE157103 dataset and found that some neutrophil subtypes, such as C4, were positively correlated with the SOFA score (S8B).

Considering the abundance of the C4 subtype (namely C4-Ab) in patients with severe disease, we used the area under the receiver operating characteristic curve (AUC) analysis to evaluate its diagnostic performance for severe COVID-19 prediction. The overlap of detected TFs between pySCENIC and DEGs analyses (Figure 6C) also served as a TF module predictor, including upregulated IRF7 and downregulated FOS/JUNB. In the GSE171110 dataset, the TF module displayed remarkable performance in predicting severe COVID-19 (AUC = 0.88) compared to the C4-Ab predictor (AUC = 0.67), while the C4-Ab showed the highest performance (AUC = 0.7) compared to the TF module (AUC = 0.66) in the GSE157103 dataset (Figure 7). Collectively, the percentage of the C4 neutrophil subtype and TF module exhibited good or moderate diagnostic performance for distinguishing severe COVID-19 from healthy status in the two-validation bulk RNA-seq datasets.

## 3. Discussion

The SARS-CoV-2 virus is responsible for COVID-19, an extremely contagious respiratory infection. Several studies have shown that neutrophils are activated in response to viral infections. Neutrophils play a critical role in the immune system and can help fight infections and inflammation-induced injuries. Moreover, neutrophil phenotypes are altered and plastic in response to the pathogenesis and progression of many diseases. Studies on the phenotypic and functional diversities of neutrophils help us understand the role of neutrophils in antimicrobial functions and pathogenic immune processes. However, the functional descriptions of the various neutrophil phenotypes after SARS-CoV-2 infection are still not fully understood.

This study identified and validated eight neutrophil subtypes, including pro-neutrophil and pre-neutrophil, immature neutrophil, and five mature neutrophil subtypes. We demonstrated that these neutrophil subtypes showed common and/or diverse functions by analyzing scRNA-seq, bulk RNA-seq, and proteomic datasets. The identified neutrophil subtypes were characterized by distinct features, including various numbers of activated signatures, different representative signaling pathways, and multiple enriched molecular functions. Some subtypes related to healthy cells possess multiple functions, such as enriched ribosome biosynthesis and transcription/translation activity. Some genes that were associated with virus-infected cells showed significant enrichment in immune responses, metabolic processes, and various signaling pathways. These findings suggest that neutrophils show various phenotypic and functional characteristics and that such a wide variety of neutrophil subpopulations may lead to diverse responses to diseases. For example, C4 neutrophils (that is, pro-neutrophils) are associated with severe and fatal diseases. Numerous activation signatures (42 genes) were detected, showing considerable enrichment in neutrophil-mediated immunity and some signaling pathways (Figure 3). CCC analysis further indicated the presence of a representative CXCL signaling pathway (i.e., the L–R pair of *PPBP* and *CXCR2*) in the C4 population (Figure 4C and Figure 5A). The *PPBP* ligand, one of the specific signatures of the C4 subtype in this study, is involved in various conditions; *PPBP* was detected in activated platelets that are involved in chronic inflammation during the development of atherogenesis and coronary heart disease (CHD), and thus could be used as a biomarker for CHD risk in postmenopausal Thai women [58]. Combined with *PADI4* expression, *PPBP* expression may be considered a non-invasive multi-marker approach offering diagnostic and clinical value in patients with suspected lung cancer [58]. Another study demonstrated that *PPBP* is a major repressive target of glucocorticoids that promote gluconeogenesis [59,60]. Interestingly, we found that the glycolysis/gluconeogenesis pathway was enriched in the C4 subtype (Figure 3E). One current study has reported that glucocorticoids (dexamethasone) impact neutrophil/platelet degranulation and were extensively utilized during the second wave of the COVID-19 pandemic, but glucocorticoids are not completely effective across the diverse dysregulations caused by COVID-19 infection [61]. Our study provided evidence that glucocorticoid treatment is not fully effective due to the heterogeneity of neutrophils. Additionally, the transmembrane receptor expression of *CD45* and *CAP1* was detected in the C4 fraction. Our phenotypic and functional descriptions of the C4 subtype may provide insights into the specific responses in severe and fatal cases. Moreover, we found that the cluster-21 neutrophils with unknown function, from immune cell expression clusters in the human protein atlas dataset (HPA; https://www.proteinatlas.org/, accessed on 15 November 2023), were associated with the C4 subtype that we described, as they showed an overlap of signatures of *PPBP* and several defensin genes (i.e., *DEFA1/3/4*).

Similar to *PPBP*’s specific association with C4, *SORT1*, *RETN*, and *GRN* were unique to C7 (pre-neutrophils) (Figure 5 and Table S2). These genes also exhibited high expression in monocyte-related cells in the HPA dataset, such as non-classical and intermediate monocytes. These may potentially provide some evidence for a previous study that pre-neutrophils have the functional attributes of transitional pre-monocytes [13]. Together with our observation of ANXA1 in C4 and C7 neutrophils and their functions of recruiting monocytes, these findings may potentially shed light on the mechanism of how pro-neutrophils or pre-neutrophils complexly interact with monocytes during COVID-19 disease. Beyond that, the abundance of the C1, C4, and C7 subtypes in patients with COVID-19, coupled with the enriched type I interferon pathway in C1 (mature neutrophils), may imply the probable joint function between these immature and mature neutrophils in responding to SARS-CoV-2 infection.

However, owing to the small size of the activation signatures, our study failed to describe the phenotypic and functional features of the C3 and C6 subtypes. Compared to the smallest total number of counts and genes across the eight neutrophil subtypes, the values of the C6 subtype were intermediate (Figure S9). Together with the relatively large number of cells of these two subtypes (Figure 2A,B), we hypothesized that C3 and C6 may be basal populations, which share most features with other neutrophil subpopulations. In addition, we obtained discrepant results regarding the correlation between neutrophil subtype fractions and SOFA scores. For example, the C8 population was found to be activated post-viral infection, and many signatures were observed (137 genes) using scRNA-seq data; however, this fraction negatively correlated with the SOFA score using bulk RNA-seq data (Figure 2A,B, Figure 3A and Figure S8). Therefore, increasing the size and type of datasets may help better interpret such results in the future. Moreover, future analyses, in combination with the assessment of other diseases and further investigation of key genes in the clinic (e.g., PPBP), may provide a comprehensive understanding of the heterogeneity of neutrophils and alterations in their phenotypes and functions.

## 4. Materials and Methods

### 4.1. Data Collection

Single-cell RNA sequencing (scRNA-seq) data were acquired from the COVID-19 Cell Atlas [11] and included healthy individuals and patients with different severity levels of COVID-19. The World Health Organization (WHO) score was used to evaluate disease severity as follows: healthy (WHO score 0), mild (WHO score 1–3), moderate (WHO score 4–5), severe (WHO score 6–7), and fatal (WHO score 8). scRNA-seq data were used to extract neutrophils and characterize the heterogeneity of their phenotypes and contributions to the disease. Two bulk RNA sequencing (bulk RNA-seq) datasets with accession numbers GSE157103 [7] and GSE171110 [30], and one human plasma proteome dataset (GSE207015) [62] were downloaded from the Gene Expression Omnibus (GEO) database to validate the identified neutrophil phenotypes in the scRNA-seq data and evaluate the performance of disease prediction models for severe COVID-19. For GSEA157103, the Sequential Organ Failure Assessment (SOFA) score was used to define the disease severity. Nine healthy individuals and 56 patients with COVID-19 were selected for the validation analysis. For the GSE171110 cohort, ten healthy and 44 severely ill samples were used. For the GSE207015 dataset, 41 healthy donors and 52 patients with different severities of COVID-19 were assessed. Additional details on the clinical and sample characteristics are presented in Table S1.

### 4.2. scRNA-seq Data Analysis and Neutrophil Subtype Identification

Neutrophils were extracted from public blood processed scRNA-seq data, which included healthy individuals and patients with different severities of COVID-19. Cell types in this dataset, such as neutrophils and other cell types, were annotated by the original study. The obtained subset was processed using the Seurat v4 package [37] in R (version 4.1.1). for normalizing gene expression, dimensionality reduction, clustering, visualizations, etc. To perform dimensional reduction analysis, the scaled data and highly variable genes (HVGs) were passed to the RunPCA function for principal component analysis (PCA). The FindNeighbors and FindClusters functions were used to cluster cells by applying a graph-based clustering approach. Clusters of neutrophils were visualized using a uniform manifold approximation and projection (UMAP) plot. A total of 3000 HVGs and thirty principal components were used in this study. For the clustering analysis, the resolution parameter, which indirectly affects how many clusters can be identified, varied from 0.1 to 1, with an increment of 0.1 for each subsequent value. We then used Clustree (version 0.5.0) [38] to visualize cell movement and detected clusters at multiple resolutions. The optimal resolution was jointly determined by further identification of markers and representative pathways. Eight clusters (neutrophil subtypes) were used for further analyses. Additionally, the ROGUE, an approach of assessing the purity of single-cell population using an entropy-based metric, was used to accurately quantify the purity of the identified eight neutrophil subtypes [39].

To identify signatures among the eight subtypes, the FindAllMarkers function was used with the following settings: min.pct = 0.25, logfc.threshold = 1, test.use = “wilcox” (a Wilcoxon rank-sum test). Finally, the signatures were decided with the additional filtering of adjusted *p* value ≤ 1 × 10^−10^ (based on the Bonferroni correction using all genes in the dataset). When comparing patients with severe COVID-19 to healthy individuals, the FindMarkers function was used with min.pct = 0.25, test.use = “wilcox”, and logfc.threshold = 0.25. Then, the differentially expressed genes (DEGs) were selected with an additional cutoff of adjusted *p* value ≤ 0.1and |avg_log2FC| ≥ 1. Collectively, the final neutrophil subtypes were identified by comparing multiple factors, such as the Clustree visualization, FindAllMarkers function, markers from the literature, ROGUE value calculation, etc. Immature and mature neutrophils were discriminated by their expression of specific markers (immature: CXCR2–MME–CD16mid; mature: CXCR2+MME+CD16hi).

### 4.3. Functional Enrichment Analysis

To describe the phenotypes and functions of neutrophil subtypes, we performed Gene Ontology (GO) and Kyoto Encyclopedia of Genes and Genomes (KEGG) pathway enrichment analyses using clusterProfiler (version 4.8.2) [63]. GO enrichment analysis was performed based on biological process (BP), cellular component (CC), and molecular function (MF). Gene Set Enrichment Analysis (GSEA) was performed to uncover the mechanisms of COVID-19 pathogenesis using C7 immunologic signature gene sets from the Molecular Signatures Database (MSigDB; https://www.gsea-msigdb.org/gsea/msigdb, accessed on 5 December 2022). The enriched pathway was filtered by a cutoff *p*-value ≤ 0.05.

### 4.4. Cell–Cell Communication Analysis

CellChat (version 1.6.1) [64] was used to explore cell–cell communications (CCC) among neutrophil subtypes and other cell types. A secreted signaling pathway database, including ligand–receptor (L–R) pairs, was used to infer cellular interactions. The label of neutrophil subtypes per cell was passed to the corresponding cells in the original scRNA-seq data to investigate cell–cell interactions among/within neutrophil subtypes and other cell types. Biologically significant signaling changes were selected to visualize communication patterns and networks.

### 4.5. Gene Regulation Inferences

pySCENIC, a python implementation of the Single-Cell rEgulatory Network Inference and Clustering (SCENIC) pipeline, was used to infer transcription factors (TFs) and their direct target genes from scRNA-seq data [65].

### 4.6. Cellular Deconvolution and Severe Disease Predictions

The CIBERSORTx [66] approach was used to estimate the proportion of cell types in bulk RNA-seq datasets. Two references (i.e., public and customized references) were used for this analysis: modified public expression signatures (LM22) and the signatures of blood cell types with the identified eight neutrophil subtypes. The original LM22 reference data, provided by CIBERSORTx, included the signature expression of 22 mature human hematopoietic populations from blood. We modified this reference by merging subtypes or similar types of cells into 12 main cell types using an average value. For the customized reference, the average expression of the detected signatures was calculated. For the severe disease prediction, receiver operating characteristic (ROC) curves were used to evaluate the performance of several predictors of severe COVID-19.

### 4.7. Cell Senescence Status Analysis

Multiple factors, including oncogene activation, DNA damage, tissue damage, aging, and viral infections, can trigger senescence [56,67]. Thus, to study the cell senescence status of neutrophil subtypes, three gene sets were used. One of these was the human aging gene set, which included 307 genes downloaded from the aging gene database (https://genomics.senescence.info/genes/human.html, accessed on 9 December 2022). The other two gene sets were acquired from the MsigDB, which were gene sets of Fridman senescence UP (77 genes) [68] and Reactome cellular senescence (214 genes). The cell senescence status of neutrophil subtypes was evaluated according to the following steps: (1) for a cell, the average expression of all genes in a senescence gene set was calculated; (2) based on the above average values, all cells from a subtype were ranked, and then the top 10% of cells were defined as senescent cells; (3) for each subtype, the relative senescent cell percentage was calculated by dividing the number of senescent cells (top 10% of cells in a subtype) by the total cell number in this subtype [69]. The percentage of senescent cells was used to evaluate the cellular senescence status of the eight neutrophil subtypes.

### 4.8. Statistical Analysis

Two-sample variables were estimated using the non-parametric Wilcoxon rank-sum test. Significant levels are represented in the violin plot as * *p* value < 0.05; ** *p* value < 0.01; *** *p* value < 0.001. All analyses were conducted using R (version 4.1.1).

## Data Availability

All the datasets can be downloaded from the indicated databases/websites. The names of the databases/websites and accession numbers (s) can be found in the article/Supplementary Materials.

## Supplementary Materials

The supporting information can be downloaded at: https://www.mdpi.com/article/10.3390/ijms25073841/s1.
