# Supplementary material for: Multi-Omics Profiling Reveals Phenotypic and Functional Heterogeneity of Neutrophils in COVID-19
Source: Int J Mol Sci. 2024 Mar 29;25(7):3841. doi: 10.3390/ijms25073841 (PMC11011481; doi:10.3390/ijms25073841)
Supplement: Supplementary file 1 [file ijms-25-03841-s001.zip › Supplementary Figures.pdf]

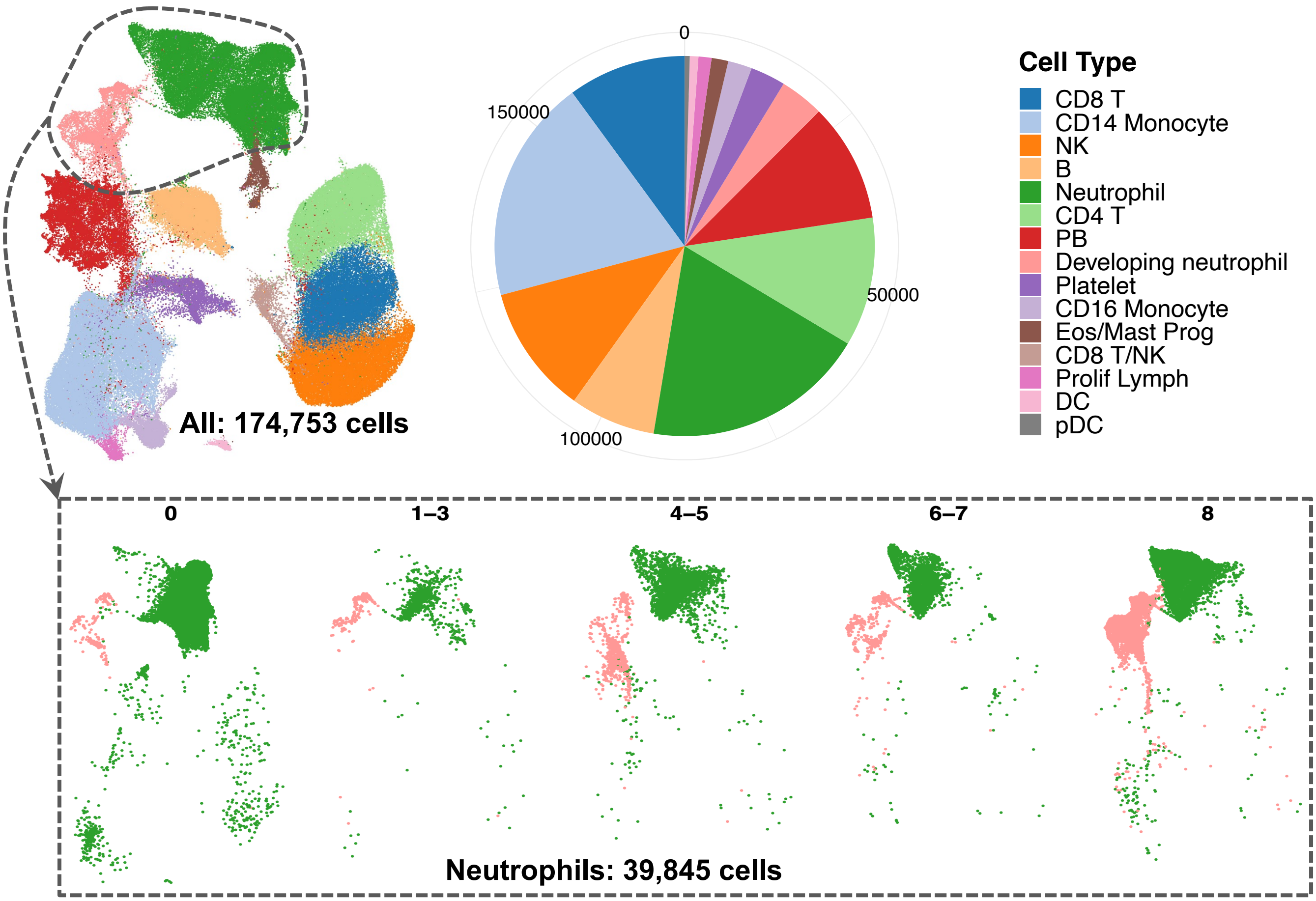

Supplementary Figure S1. Characterization of the source dataset.
The cells from the source scRNA-seq dataset were visualized using a UMAP plot. Fifteen cell types were identified in this study. A subset of cells (neutrophils only) was extracted and visualized according to COVID-19 severity. Five severity groups were defined as follows: healthy (0), mild (1–3), moderate (4–5), severe (6–7), and fatal (8).

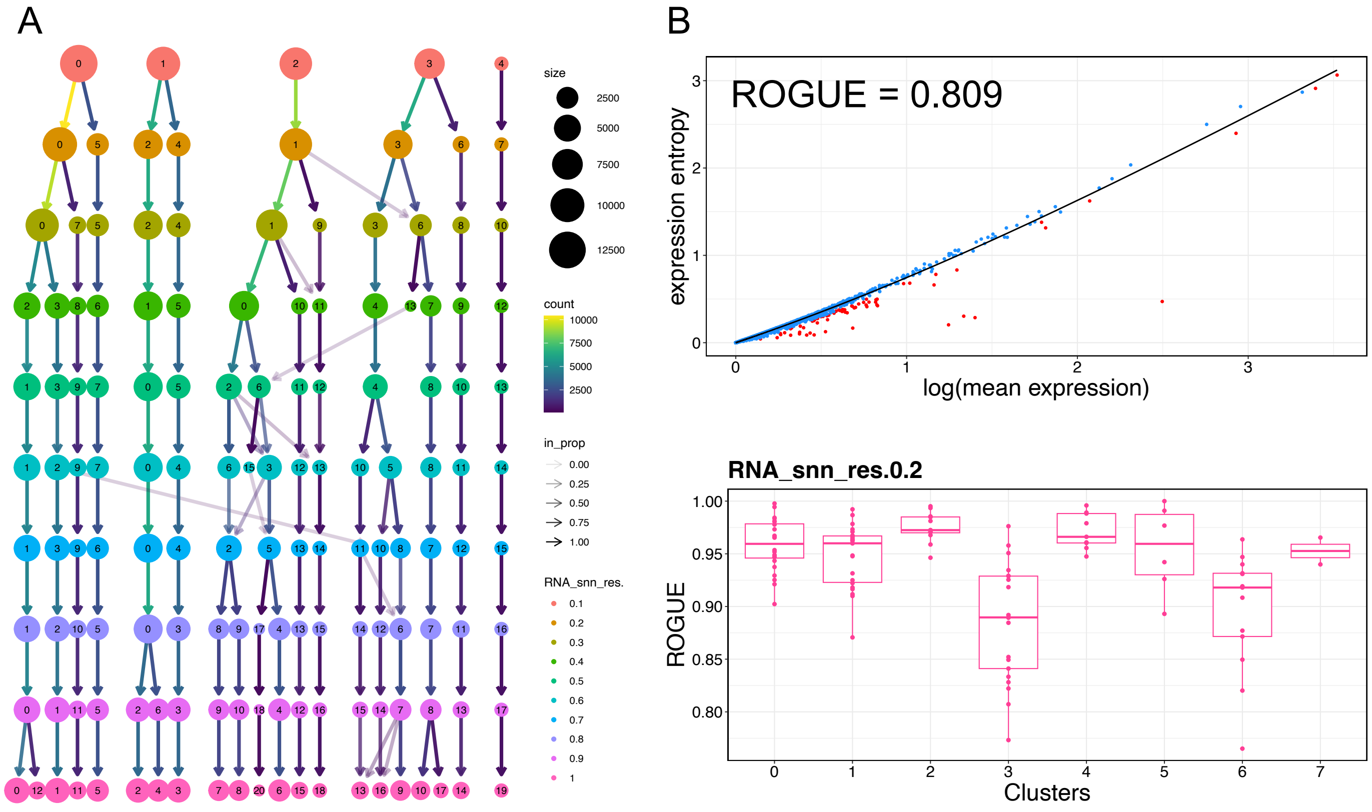

Supplementary Figure S2. Identification and quality evaluation of neutrophil subtypes. (A) Visualization of cell movement in terms of multiple resolutions. Clustree was used to visualize and define the optimal resolution for cell clustering. For clustering, the resolution parameter varied from 0.1 to 1, with an increase of 0.1 for every step. (B) Quantification of the purity of the identified subtypes using ROGUE. Top panel: the S–E plot and corresponding ROGUE value of neutrophils. The low ROGUE value (~0.8) suggested heterogeneity of neutrophils from the scRNA-seq dataset. Bottom panel: purity evaluation of putative clusters at the RNA_snn_res.0.2 resolution, with each point representing a donor. The relatively high ROGUE values for the clusters demonstrated that they were all highly homogeneous neutrophil subtypes.

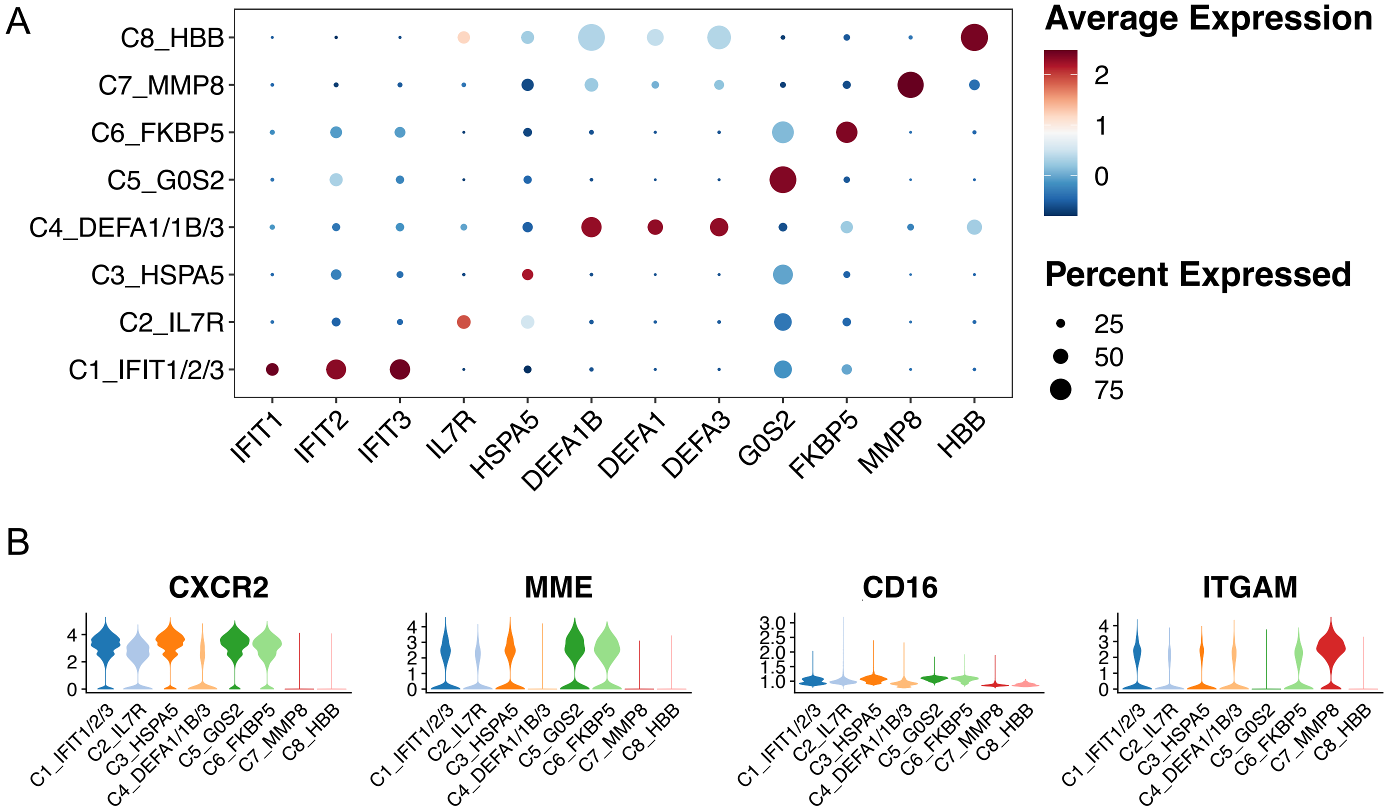

Supplementary Figure S3. Expressions of markers for each neutrophils subtype.
(A) Dot plot represents the selected subtype-specific markers that were identified by using the FindAllMarkers in Seurat. (B) Violin plots represent expressions of well-known markers for different neutrophil compartments.

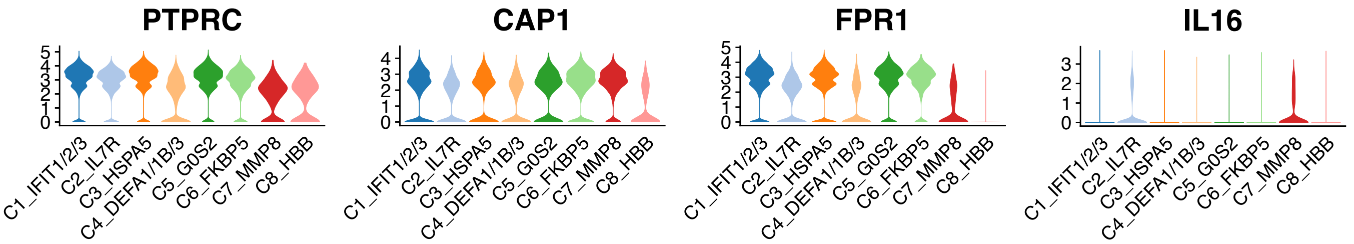

Supplementary Figure S4. Expressions of selected ligands and receptors.
Violin plots represent the expression of selected ligands and receptors, as shown in Figure 5. Significant gene expression analysis was performed by comparing a relative subtype to all the rest of subtypes. These genes were not identified by using FindAllMarkers in Seurat.

A

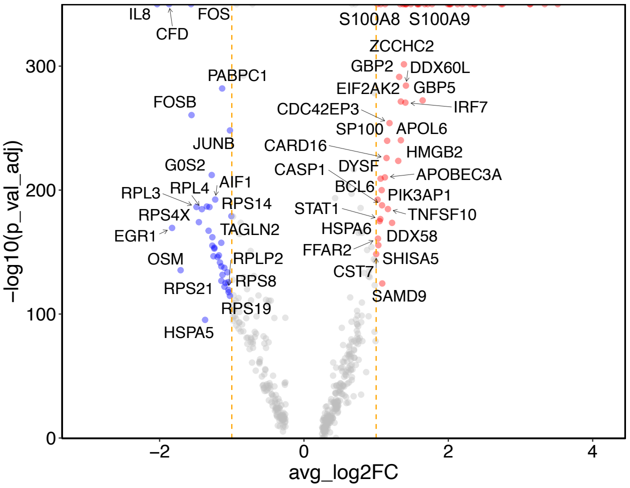

B

DEGs	Enriched pathways
Up	• NOD-like receptor signaling pathway<br>• Influenza A<br>• RIG-I-like receptor signaling pathway<br>• Necroptosis<br>• IL-17 signaling pathway<br>• Toll-like receptor signaling pathway<br>• C-type lectin receptor signaling pathway
Down	Ribosome

Supplementary Figure S5. Comparisons between severe patients and healthy donors. (A) Volcano plot represents the DEGs in patients with severe COVID-19 compared to healthy individuals. Red, blue, and grey colors represent upregulated, downregulated, and stable genes in severe COVID-19 cases, respectively. (B) Identification of enriched pathways using DEGs in (A).

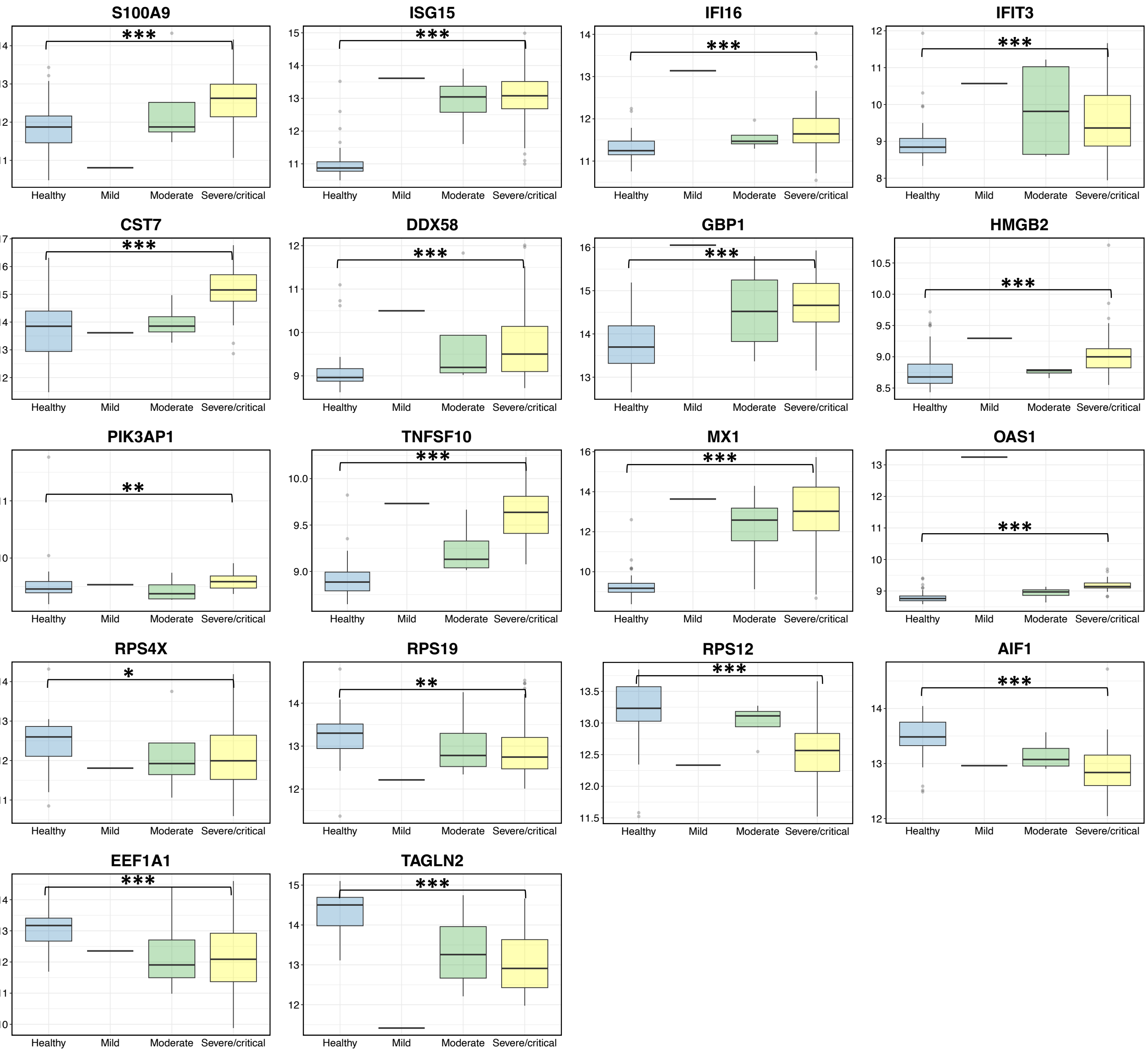

Supplementary Figure S6. Visualizations of protein abundant levels in plasma proteome dataset.

For GSE207015, the COVID-19 severity was classified as mild, moderate, severe, and critical. The x axes correspond to the COVID-19 severity of samples. Shown are significant levels by comparing healthy and severe/critical samples. * p value < 0.05; ** p value < 0.01; *** p value < 0.001.

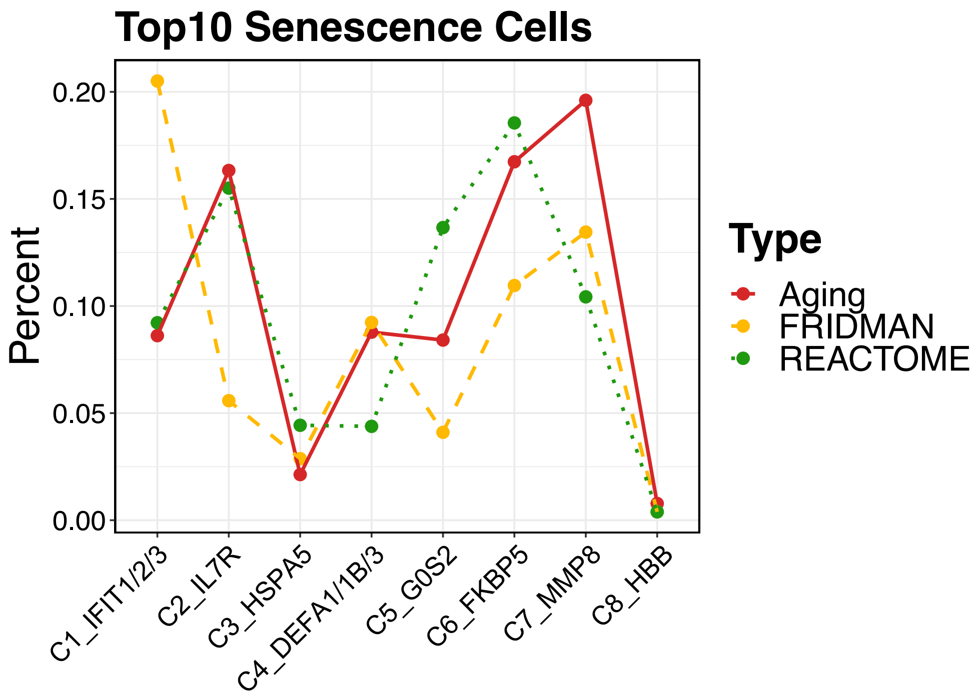

Supplementary Figure S7. Cell senescence status across the eight neutrophil subtypes. The percentage of senescent cells in each cluster was calculated. Senescent cells were defined based on the average expression of the three gene sets (i.e., aging, Fridman, and reactome). The top 10% of cells in a given cluster were defined as senescent based on average expression levels.

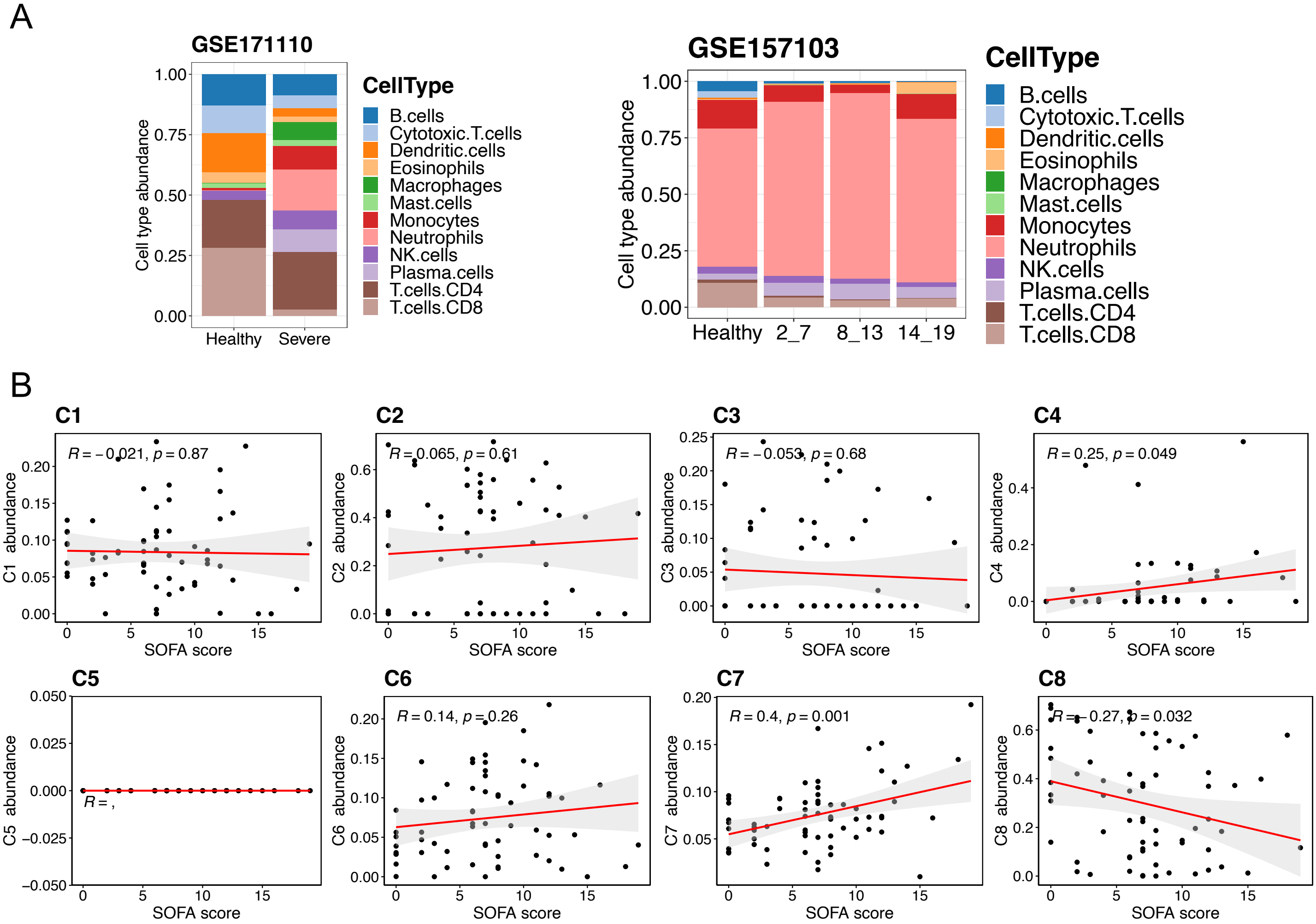

Supplementary Figure S8. Immune cell type abundance and correlation between neutrophil subtypes and SOFA score.
(A) Cell type abundance estimation for GSE171110 and GSE157103 datasets using modified LM22 reference. (B) Pearson correlation between fractions of the eight neutrophil subtypes and SOFA scores using GSE157103 dataset. SOFA: sequential organ failure assessment.

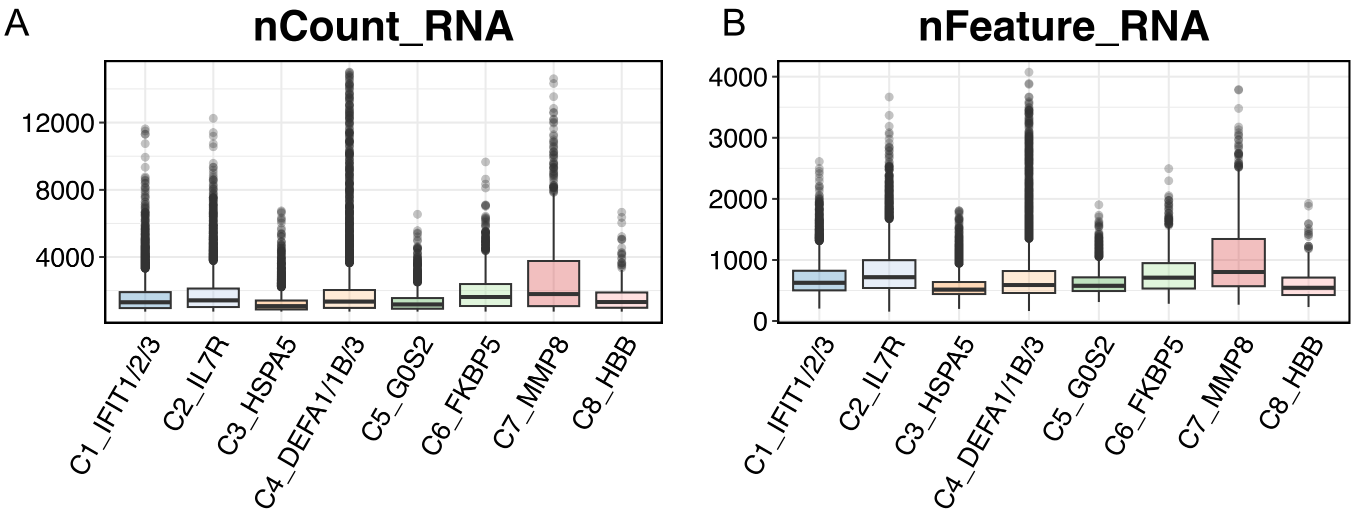

Supplementary Figure S9. Statistics of the eight characterized subtypes of neutrophils. For each cell, box plots indicate total numbers of (A) UMI counts and (B) gene across the defined eight subsets of neutrophils, respectively.
